# Shift toward greater pathologic post-myocardial infarction remodeling with loss of the adaptive hypertrophic signaling of alpha1 adrenergic receptors in mice

**DOI:** 10.1371/journal.pone.0188471

**Published:** 2017-12-07

**Authors:** Che-Chung Yeh, Yanying Fan, Yanchun Xu, Yi-Lin Yang, Paul C. Simpson, Michael J. Mann

**Affiliations:** 1 Cardiothoracic Translational Research Laboratory, University of California San Francisco, San Francisco, California, United States of America; 2 Division of Cardiology, Veterans Affairs Medical Center and University of California San Francisco, San Francisco, California, United States of America; University of Cincinnati College of Medicine, UNITED STATES

## Abstract

**Rationale:**

We have hypothesized that post-infarction cardiac remodeling can be influenced by shifts in the balance between intracellular mediators of “pathologic” and “physiologic” hypertrophy. Although alpha1 adrenergic receptors (alpha1-ARs) mediate pro-adaptive hypertrophy during pressure overload, little is known about their role or downstream mediators after myocardial infarction.

**Methods:**

We performed loss-of-function experiments via coronary ligation in alpha1A-AR knockout (AKO) mice. Post-myocardial infarction (MI) remodeling was evaluated via echocardiography, quantitative reverse transcription-polymerase chain reaction (RT-PCR) analysis of cardiac fetal gene expression, histologic analysis of myocyte size, post-MI fibrosis and apoptosis, and Western blot analysis of apoptotic regulators.

**Results:**

Alpha1A-AR knockout paradoxically increased post-MI hypertrophy compared to wild type controls (WT), but also increased ventricular dilatation, fibrosis, apoptosis, and 4-week post-MI mortality (64% in AKO vs. 25% in WT, P = 0.02), suggesting a shift toward greater pathologic hypertrophy in the absence of pro-adaptive alpha1A effects. alpha1A-AR knockout increased phospho-p38 levels in the pre-MI myocardium compared to WT (0.55 ± 0.16 vs. 0.03 ± 0.01, P<0.05) but decreased phospho-ERK1/2 post-MI (0.49 ± 0.35 arbitrary units vs. 1.55 ± 0.43 in WT, P<0.05). Furthermore, expression of pro-apoptotic factor Bax was increased (1.19 ± 0.15 vs. 0.78 ± 0.08, P<0.05) and expression of anti-apoptotic factors Bcl2 was decreased (0.26 ± 0.01 vs. 0.55 ± 0.06, P<0.01) compared to WT.

**Conclusions:**

Alpha1A-AR provides an important counterbalance to pathologic pathways during post-MI remodeling that may be mediated through ERK1/2 signaling; these observations provide support for further development of an alpha1A-AR/ERK-based molecular intervention for this chronic, often fatal disease.

## Introduction

We have hypothesized that a dynamic balance exists between signaling pathways during the heart’s response to various stresses, with pro-adaptive pathways dominating in the context of physiologic hypertrophy (e.g. in response to exercise or pregnancy), and greater mal-adaptive signaling resulting in eventual decompensatory changes (e.g. in pressure overload or post-infarction cardiomyopathy) [1,2]. Such a dynamic balance creates an opportunity for more precise design of molecular interventions for progressive, often intractable heart failure, one of the largest single sources of morbidity and mortality in the United States.

The adrenergic signaling system can influence both physiological and pathological cardiac hypertrophy, particularly via the stimulation of different adrenergic receptors (ARs). Chronic administration of a beta-AR agonist to rats with compensated concentric hypertrophy has implicated beta-AR signal activation in the development of pathological hypertrophy, while activation of G-protein-coupled alpha1- adrenergic receptors (alpha1-ARs) has been linked to physiological hypertrophy [3,4]. It is well documented that three alpha1-AR subtypes, 1A/C, 1B and 1D, are expressed in cardiovascular tissues and play important roles in the regulation of various physiological process, such as cardiac contractility, blood pressure regulation, and the growth and proliferation of vascular smooth muscle cells [5–7].

Two of the alpha1-AR subtypes are predominantly expressed in the heart, alpha1A and alpha1B, and both have been shown to regulate cardiac contractility via suppression of Ca2+ transients, increased myofilament Ca(2+)-sensitivity, and altered myofilament function [8,9]. Studies using the alpha1A knockout mouse model also showed that these specific receptors are required for physiological cardiac hypertrophy [10]. The deletion of both alpha1A and alpha1B receptors resulted in an exacerbation of the maladaptive response to pressure overload, with increases in apoptosis, dilated cardiomyopathy, and death, suggesting that they might play an ameliorative role counterbalancing other pathologic stimulation. Among the alpha1-AR subtypes, alpha1A-ARs have been demonstrated to play a particularly important role in ischemic preconditioning, and in suppression of cardiac myocyte apoptosis and activation of pro-survival mitogen activated protein kinase (MAPK) signaling [11–13]. Furthermore, gain-of-function studies have documented that enhanced inotropy from transgenic alpha1A-AR overexpression prevents remodeling of the left ventricle (LV), preserves function, and reduces acute heart failure death after MI or induction of pressure overload [14,15].

Studies from our laboratory and others’ have indicated that numerous members of the mitogen activated protein (MAP) kinase family play important roles in the remodeling of left ventricle after myocardial infarction (MI) [2,16,17]. Earlier studies indicated that the alpha1A-AR is the primary receptor subtype responsible for AR-induced activation of pro-survival and pro-adaptive MAP kinases MAP kinase/extracellular signal-related kinase (ERK) kinases 1/2 (MEK1/2)-ERK1/2 in the myocardium [13,18]. We have postulated that the heart possesses a repertoire of responses to various forms of stress, and that the characteristic reversible, adaptive physiologic responses to certain stresses (e.g. exercise or pregnancy) are regulated by a differential balance of signaling factors compared to maladaptive pathologic responses to others (e.g. MI or pressure overload). In this study, we hypothesized that in the absence of adaptive hypertrophic signaling mediated by the alpha1A-AR, an increase in the balance favoring pathologic signaling would lead to a decline in pro-adaptive, physiologic remodeling after MI, and subsequently to a corresponding exacerbation of pathologic post-MI remodeling. We examined patterns of pathologic remodeling post-MI in the alpha1A-AR knockout mouse (AKO), including functional/structural changes, changes in patterns of fetal gene expression, and changes in apoptosis signaling and apoptosis rates.

## Methods

### Alpha1A-AR knockout model and mouse coronary artery ligation

The global alpha1A-AR knockout mouse line was generated by Dr. Simpson’s laboratory and its phenotype has been described previously [19]. The AKO mouse has previously been found to be normotensive, and no obvious phenotypic changes have been observed in unstressed AKO mice [20]. Wild type (WT) and AKO male mice (8–10 weeks old) were anesthetized with 1.5% isoflurane using a rodent ventilator (Harvard) at 115 breaths/min. A left lateral thoracotomy incision was placed at the level of the fourth interspace and a 7.0 polypropylene suture was used to ligate the left anterior descending artery (LAD). A sodium hyaluronate-carboxymethyl cellulose membrane patch (Seprafilm®, Genzyme) was placed between the left ventricle and chest wall to facilitate reoperation. The left chest was then closed and the animal was recovered in a light-warmed incubator after extubation. Buprenorphone (0.1 mg/kg) was administered every 4–6 hours for evidence of pain. All animals were housed in sterile rodent microisolator caging under standard rodent husbandry conditions, and all procedures conformed strictly with the *Guide for the Care and Use of Laboratory Animals* published by the US National Institutes of Health (NIH Publication No. 85–23, revised 1996), and were approved by the Institutional Animal Care and Use Committee of University of California, San Francisco (UCSF IACUC approval # AN107727-02). All surgery was performed under isoflurane anesthesia to minimize cardiodepressive effects, and all efforts were made to minimize suffering. All animals were monitored at least twice daily for signs of distress; animals demonstrating progressive weight loss, diminished food intake, lethargy or inactivity were monitored more frequently. Animals showing signs of extensive wasting (>15% weight loss), inactivity, labored breathing weakness or slow movement were euthanized within 12 hours via pentobarbital injection (75 mg/kg, i.p.). Personnel involved in animal experiments underwent training in the observation, recognition, and documentation of the intervention point parameters, and in the humane handling of laboratory animals, and all had over 7 years experience in rodent care.

### Treatment of post-MI remodeling with captopril

Where indicated in the text, the angiotensin converting enzyme (ACE) inhibitor captopril (West-ward Pharmaceutical Corporation, Eatontown, NJ) was administered in the drinking water (2g/L) of randomly selected animals from the time of LAD ligation until sacrifice of the animal at 4 weeks post-MI [21]. Drinking water was changed 3 times per week. This dosing regimen has not been associated with changes in blood pressure in past studies [21].

### Echocardiography

Transthoracic echocardiography was performed in conscious mice 4 weeks post-MI using an Acuson Sequoia 512 machine and a 13-MHz probe. A two-dimensional short-axis view of the left ventricle was obtained at the level of the papillary muscles (i.e. remote to the infarction) and two-dimensional M-mode tracings were also recorded. LV fractional shortening was calculated as (LVDd–LVDs)/LVDdX100, where LVDd = LV diastolic dimension and LVDs = LV systolic dimension. EF ejection fraction was calculated as (EDV-ESV)EDV where EDV = end diastolic volume and ESV = end systolic volume.

### Tissue harvest and Western blot

Four weeks after LAD ligation, mice were anaesthetized with pentobarbital (75 mg/kg, i.p.) prior to diastolic arrest via injection with 1 ml of 1M KCl (with 100 U heparin). Then the hearts were perfused for 10 minutes with phosphate buffered saline with 100U/ml heparin. Non-infarcted base halves of left ventricles for Western blot were snap frozen in liquid nitrogen, while other tissues were fixed in 10% paraformaldehyde for histology.

Snap frozen hearts were weighed and added to homogenization buffer containing 0.13 M KCI, 1 mM EDTA, 1mM Na3(VO4), 5 mM NaF, 20 mM HEPES and Roche Complete Protease Inhibitor, then homogenized on ice. The homogenate was centrifuged at 16,000g for 20 minutes, and the resultant supernatant was used to determinate protein concentration and kept in -80°C. Tissue lysate (10microgram/well) from each heart was separated by electrophoresis on 4–12% Bis-Tris gels. Separated proteins were then blotted to PVDF membranes. The blot was blocked by 5% non-fat milk, then incubated with primary antibody overnight at 4°C, followed by incubation with a species-appropriate horseradish peroxidase-conjugated secondary antibody (AnaSpec). Protein expression on the blots was detected by ECL plus Western blotting detection system (Amersham), and was analyzed by NIH ImageJ software.

### mRNA measurements

RNA samples were extracted from non-infarcted LV tissue with Total RNA Purification System (Invitrogen). For cDNA syntheses, 500 ng of total RNA was reverse-transcribed using M-MLV reverse transcriptase (Promega). Reactions were incubated for 60 min at 37°C according to the manufacturer's protocols. SYBR Green real-time PCR analyses of mouse alpha myosin heavy chain (alphaMHC), betaMHC, atrial natriuretic peptide (ANP) and glyceraldehyde 3-phosphate dehydrogenase (GAPDH) in the cDNA samples was conducted in an ABI 7700 Sequence Detection System (Applied Biosystems) with 5% of the RT product, primers at 100 nM and SYBR Green Master (ABI). Primers used in the PCR were alphaMHC, 5’-gtcatccagtactttgccagc-3’ and 5’-tcaatggaggccacggacac-3’; betaMHC, 5’-ttgagaatccaaggctcagc-3’; ANP, 5’-gagaagatgccggtagaagatg-3’ and 5’-gagcactgccgtctctcaga-3’; and GAPDH, 5’-catggccttccgtgttccta-3’ and 5’-cctgcttcaccaccttcttgat-3’. Relative levels of mRNA expression of each gene were normalized separately using GAPDH gene expression as loading controls.

### Histologic analysis

Five-micron sections of paraffin-embedded hearts were stained with Texas Red-conjugated wheat germ agglutinin (WGA, Invitrogen) and Sirius Red (Sigma) to assess myocyte size and fibrosis in the septal myocardium remote to the LV infarct. All acquired images were analyzed and compared using the ImageJ Software (Bethesda, MA, USA). Sections were also subjected to TUNEL staining (ApopTag Peroxidase In Situ Apoptosis Detection Kit, Chemicon, Temecula, CA), and apoptotic cells and total cells, identified via hematoxylin nuclear counterstaining, were counted in 3 sections/heart. Hematoxylin-eosin staining was used to distinguish the morphology of cells containing apoptotic nuclei on adjacent sections. The level of apoptosis is expressed as apoptotic cells as a percentage of total nuclei.

### Data analysis

Results are expressed as mean ± SEM. Mean values were compared by the unpaired 2-tailed Student’s t test for 2 groups. Sample size calculations were based on end point estimates from previous studies. Survival curves of groups were plotted using the Kaplan-Meier method and compared by the log-rank test. P-values less than 0.05 were considered statistically significant.

## Results

### Loss of alpha1A receptor modulates MAPK signaling in the pre- and post-MI myocardium

**Fig 1:**
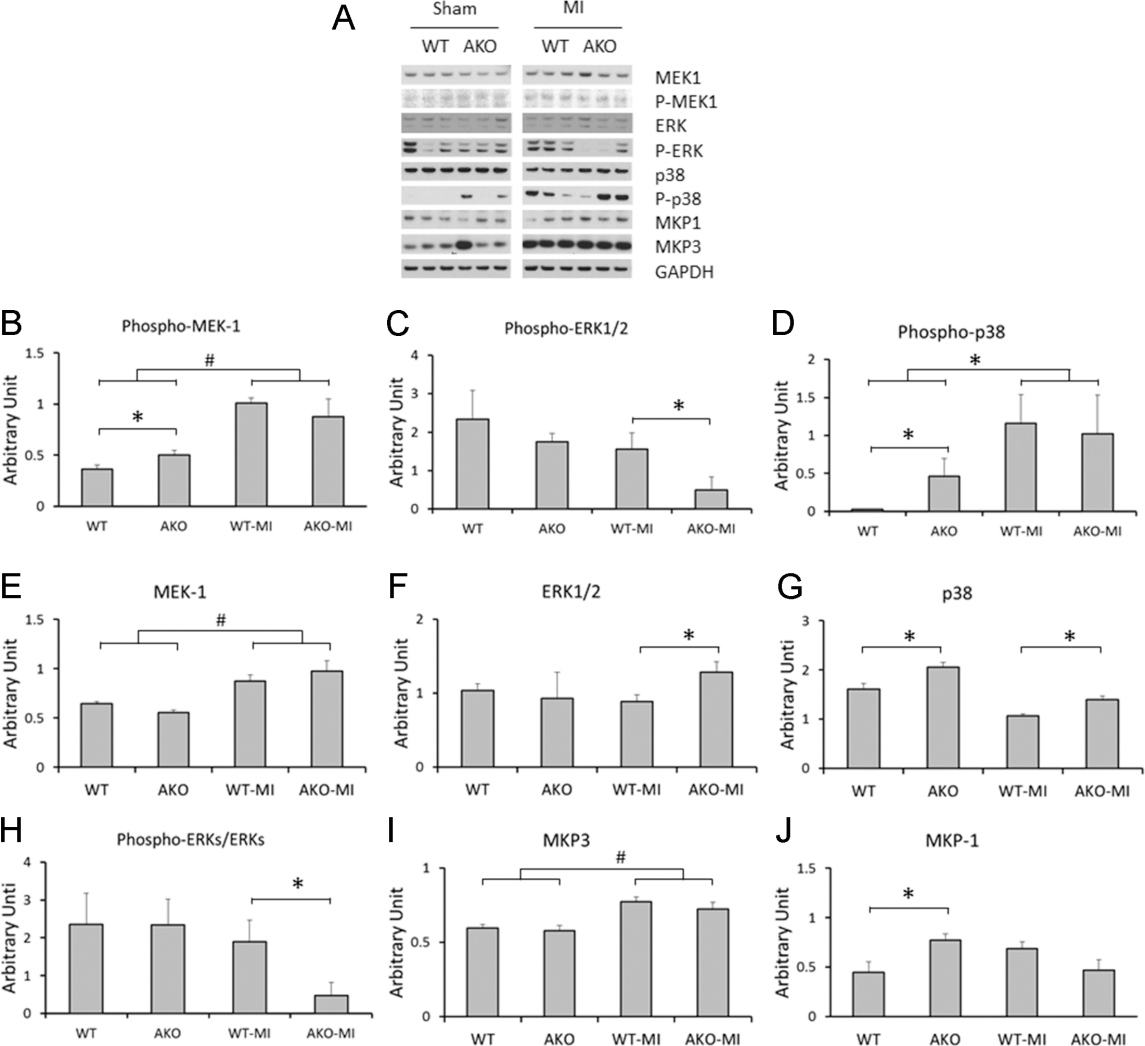
Loss of alpha1A receptor and MAPK signaling in the pre- and post-M myocardium. (A) Reprehensive Western blots of MAKP signaling molecules in the myocardium from WT(sham, n = 4), AKO (sham, n = 3), WT-MI (n = 6) and AKO-MI (n = 5). (B-I) Analyses & comparison of expression of MAPK molecules in the pre-MI (sham) and post-MI myocardium, normalized to GAPDH protein levels. *P<0.05, #P<0.01.

Pro-survival and pro-adaptive MEK1/2-ERK1/2 MAP kinase signaling has been associated with alpha1A-AR in the myocardium, and the impact of deletion of this receptor on MAPK signaling was therefore investigated. Loss of alpha1A receptor did not result in a decrease of phospho-ERK1/2 (P-ERK1/2) or of the ratio of P-ERK1/2:total ERK1/2 in sham control mice. However, there was significantly higher expression of p38 (P<0.05), associated with an increase in phospho-p38 and in its phosphatase regulator MKP1 (P<0.05), in AKO-sham myocardium compared to WT-sham myocardium (Fig 1). Myocardial infarction induced significant increases in the expression of MEK-1, phopho-MEK1, MAP Kinas phosphatase-3 (MKP3) and also phospho-p38 in the non-infarcted, remote myocardium of hearts subjected to LAD ligation, compared to the myocardium of non-MI mice from both WT and AKO controls (P<0.01, Fig 1), but this increase in MEK1 activation did not result in a significant change in downstream ERK1/2 phosphorylation. Interestingly, loss of alpha1A-AR was associated with down-regulation of phospho-ERK1/2 and a reduction in the phospho-ERK1/2: total ERK1/2 ratio in the post-MI remote myocardium (Fig 1; P<0.05). Although the expression of p38 continued to be higher in the non-infarcted myocardium of post-MI AKO mice compared to post-MI WT mice (p<0.05, Fig 1), p38 activation (i.e. phosphorylation) was not.

### Effect of loss of alpha1A receptor on LV remodeling

**Fig 2:**
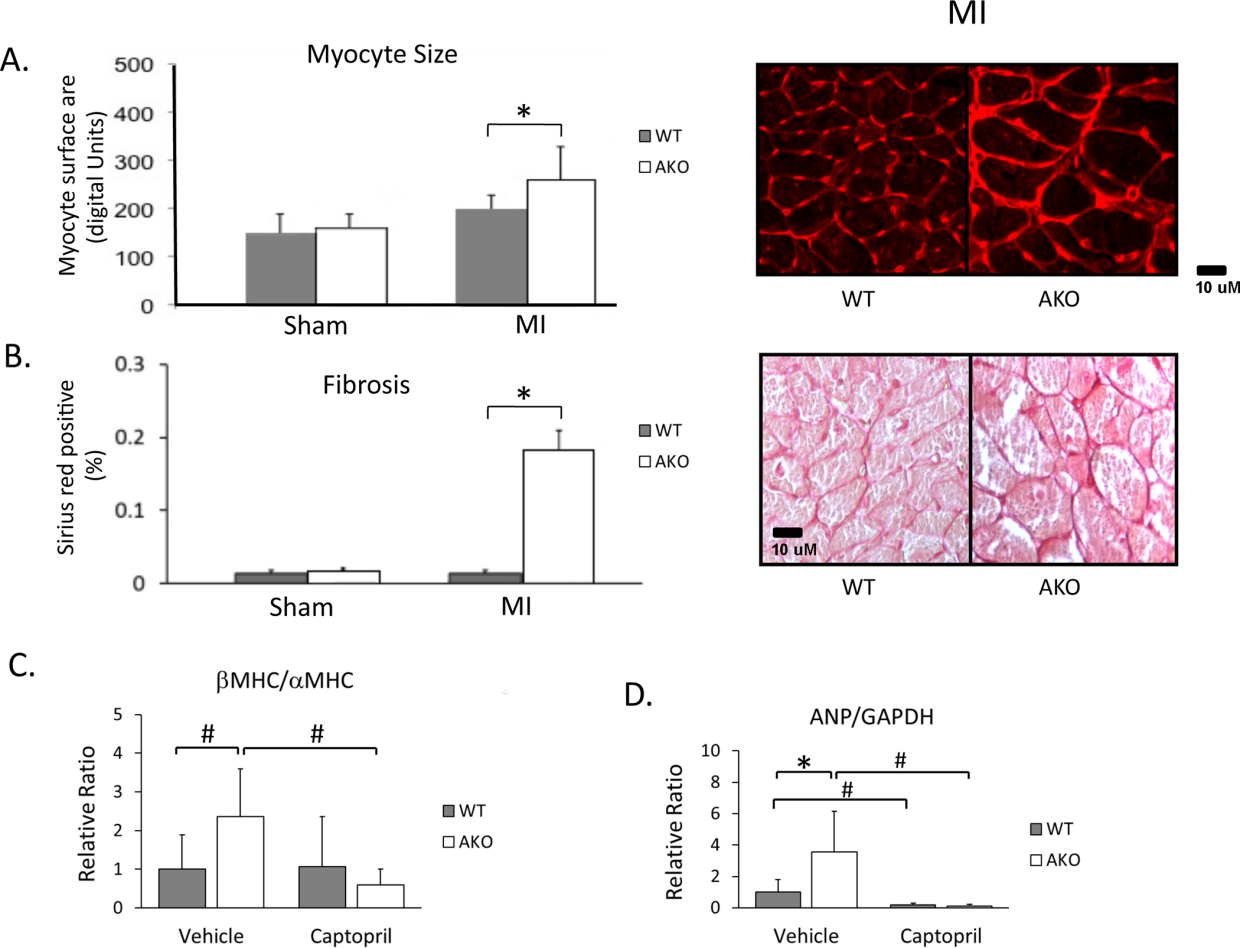
Effect of loss of alpha1A receptor on LV remodeling at 4 weeks post-MI. (A) Myocyte surface area revealed by WGA staining, n = 4–5, bar = 20 μm. (B) Fibrosis as measured Sirius Red collagen staining, n = 5–9. betaMHC/alphaMHC mRNA ratio (C) and ANP mRNA expression (D) in the 4-week post-MI myocardium. AKO mice exhibited a significantly higher betaMHC/alphaMHC ratio as well as ANP mRNA expression than WT mice, while captopril treatment eliminated the difference between them. Captopril significantly reduced ANP expression in both WT and AKO mice, but reduced the betaMHC/alphaMHC ratio only in AKO mice. Number of mice analyzed in each group: WT-vehicle = 7, AKO-vehicle = 9, WT-Captopril = 10, AKO-Captopril = 9. *P<0.05, ^#^P<0.01.

Although the alpha1-AR has been associated with the induction of physiologic hypertrophy [3, 4], the loss of alpha1A receptors was associated with a paradoxical *increase* of hypertrophy in AKO vs. WT hearts. Numeric but non-significant increases in LV mass were observed in AKO even prior to MI (Table 1), but differences in both LV mass (Table 1) and cardiac myocyte size (Fig 2A) were significant compared to WT at 4 weeks post-MI. However, these reflections of increased cardiac hypertrophy in AKO mice were also associated with increases in end systolic and diastolic volumes (Table 1), suggesting that a possible reduction in alpha1A-AR-stimulated physiologic hypertrophy led instead to an increase in pathologic hypertrophy and subsequently to worsened post-MI ventricular dilatation. These observations were further corroborated by a significant increase in the heart weight:body weight ratio of AKO mice 4 weeks after MI compared to WT controls (Table 1).

**Table 1:** Cardiac function, hypertrophy and dilatation in WT and AKO mice 4 weeks post-MI.

	WT			AKO		
	Sham	Vehicle	Captopril	Sham	Vehicle	Captopril
EF (%)	72±1	58±2	59±3	71±1	58±2*	67±1*
FS (%)	44±1	32±1	31±2	43±1	31±2*	37±1*
LVVtotal (uL)	118±11	151±11^†^	129±11	133±13	179±16*^†^	109±6*
LVEDV (uL)	50±6	70±7^†^	67±7	53±5	88±11*^†^	52±4*
LVESV (uL)	14±1	32±5^†^	29±5	15±1	42±7*^†^	17±2*
LVmass(uL)	71±6	85±4^†^	64±4	84±9	96±6*^†^	60±3*
HW:BW	5.2±0.2	6.4±0.1*^†^	5.4±0.2*	5.3±0.1	7.1±0.3*^†^	4.9±0.6*
n	5	28 (19)	10	5	28 (18)	9

*P<0.05, Vehicle vs. Captopril

^†^P<0.05 WT_Vehicle_ vs. AKO_Vehicle_.

Sham = sham operated, non-infarcted mice. () = n for HW:BW measurements.

Global ejection fraction (EF) and local fractional shortening (FS) at the level of the papillary muscles was already substantially depressed in post-MI WT hearts, and was not lower in AKO mice. Myocardial fibrosis, however, was noted to be substantially worse in the myocardium remote to the infarcts in AKO vs. WT mice (Fig 2B).

Alpha1A-AR knockout was also associated with an increase in both the betaMHC/alphaMHC mRNA expression ratio and in the mRNA expression of ANP (Fig 2C and 2D), changes that would be consistent with a pattern of increased cardiac fetal gene expression that has been linked to pathologic remodeling.

### Effect of loss of alpha1A receptor on mortality

**Fig 3:**
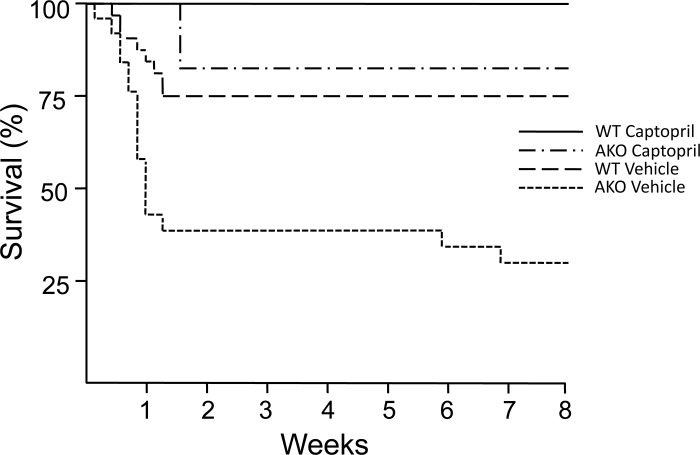
Post-MI survival of WT and AKO mice with or without captopril treatment. AKO mice experienced significantly higher mortality than WT (WT-vehicle vs. AKO-vehicle, P = 0.016). Captopril improved post-MI survival in both WT and AKO mice (WT-vehicle VS. WT-Captopril, P = 0.052; AKO-vehicle VS. AKO-Captopril, P = 0.012), and minimized the mortality difference between WT and AKO (WT-Captopril VS. AKO-Captopril, P = 0.1473). Number of mice analyzed in each group: WT-vehicle = 32, AKO-vehicle = 25, WT-Captopril = 11, AKO-Captopril = 11.

The exacerbation of post-MI remodeling in AKO mice was associated with a significant increase in mortality rate compared to that of littermate WT controls at 4 weeks post-MI (Fig 3, AKO mortality of 64%, n = 25, vs. 25% in WT, n = 32, P = 0.016). Mortality was not observed in sham controls in either the AKO or WT groups. Five animals died before reaching euthanasia criteria despite close monitoring on a twice-daily basis. In other cases, necropsy reflected changes in the lungs and liver consistent with end stage heart failure.

### Effect of loss of alpha1A receptor leads to increased pro-apoptotic signaling in the non-infarcted myocardium

**Fig 4:**
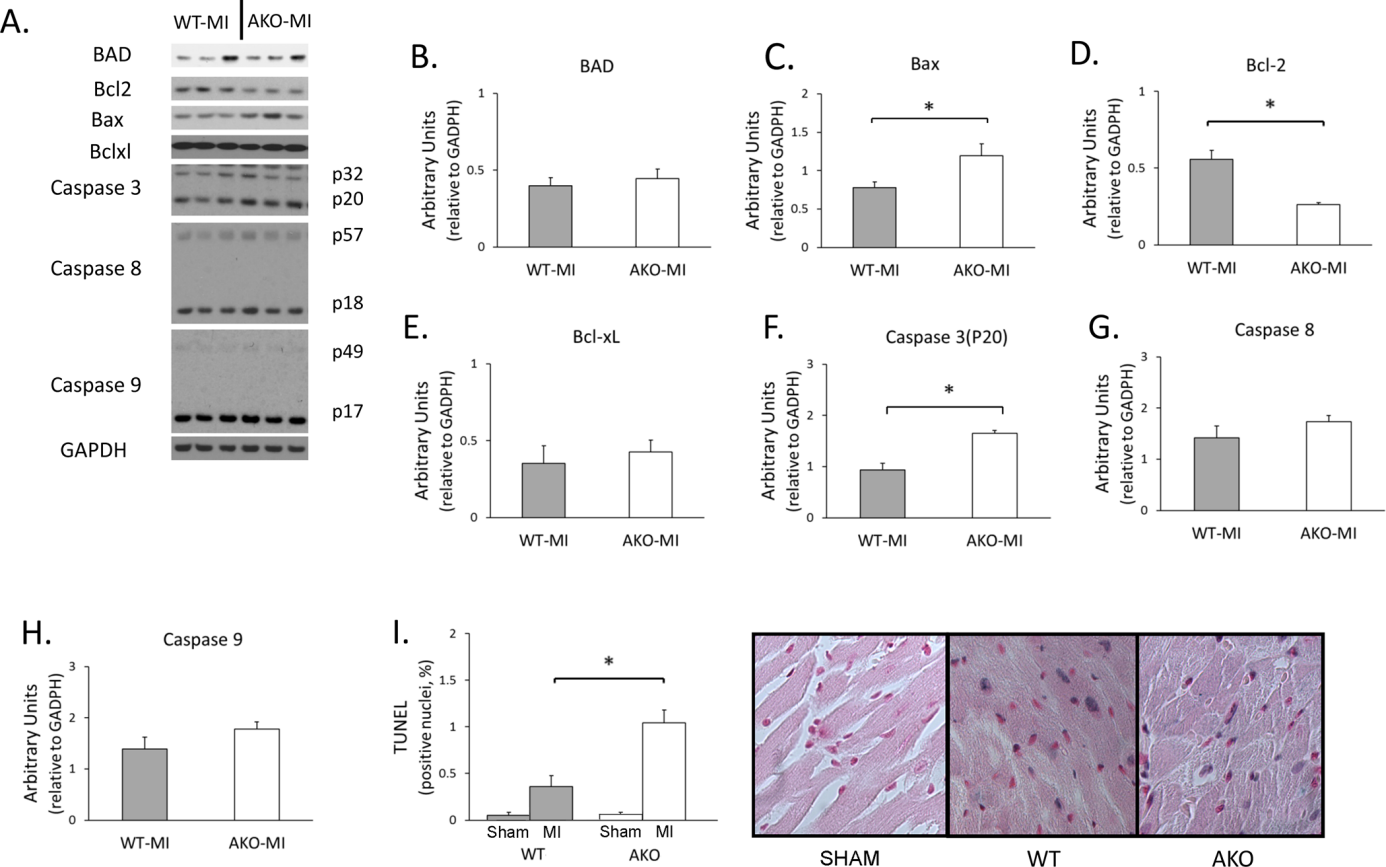
Regulation of apoptosis in the AKO myocardium 4 weeks after MI. (A) Western blots of apoptosis regulators. (B-H) Expression of apoptosis regulators in WT and AKO mice. The anti-apoptotic Bcl-2 is significantly lower in AKO than in WT mice in the non-infarcted myocardium, while the pro-apoptotic Bax and caspase 3 (p20) are significantly higher in AKO (*P<0.05). (I) TUNEL staining in the non-infarcted myocardium 4 weeks post-MI. Post-MI apoptosis rates were measured as the percentage of TUNEL-positive nuclei in the myocardium (*P<0.05); representative micrographs are presented from TUNEL-stained WT and AKO hearts. Number of mice analyzed in each group: WT-MI = 6, AKO-MI = 4.

During post-MI remodeling, cardiomyocytes in the remote, uninfarcted region experience stresses resulting both from altered ventricular geometry and from the loss of contractile function in the infracted myocardium. Increased apoptosis rates have been observed as a part of this myocardial stress response [2,21,22], and this cellular loss likely contributes to myocardial fibrosis and to an ongoing decline in compensatory function of the remaining viable myocardium. Loss of alpha1A-AR led to a significant drop in anti-apoptotic Bcl-2 protein and an increase in pro-apoptotic Bax protein in post-MI AKO hearts. Similarly, apoptosis mediator caspase 3 was increased in AKO hearts compared to post-MI WT controls (Fig 4A–4H). TUNEL staining corroborated a subsequent increase in apoptotic nuclei in the remote myocardium of these AKO hearts (Fig 4I).

### ACE inhibitor captopril reverses the effects of alpha1A-AR deficiency in the post-MI mouse myocardium

To further explore the role of exacerbated pathologic remodeling after MI in the absence of the alpha1A-AR, we investigated the effect of angiotensin converting enzyme (ACE) inhibitor captopril, known to inhibit pathologic ventricular hypertrophy, after LAD ligation. Although captopril reduced 4-week post-MI mortality in both AKO and WT mice compared to vehicle controls (Fig 3), this effect was most profound in AKO mice in which mortality was reduced from 64% to 18% (P = 0.01). Effects on mortality were related to an impact of ACE inhibition on cardiac remodeling. Although modest improvements in LV hypertrophy and dilatation were observed with post-MI captopril treatment of WT mice, more dramatic improvements in LV mass, LV end-systolic and end-diastolic volumes, and heart weight:body weight ratios were observed in the AKO hearts (Table 1). Furthermore, although local and global contractility post-MI, as reflected in measurements of FS and EF, respectively, were not lower in vehicle-treated AKO than in vehicle-treated WT mice, significant improvement was seen only in the AKO mice after ACE inhibition.

Captopril significantly decreased the expression of ANP mRNA, but not the betaMHC/alphaMHC mRNA ratio, in the remote myocardium of WT mice 4 weeks after LAD ligation (Fig 2C and 2D). However, much more dramatic changes in ANP mRNA expression and a large, highly significant decrease in the betaMHC/alphaMHC mRNA ratio was observed with captopril treatment of post-MI AKO mice (P<0.01). Interestingly, captopril inhibition of the fetal gene program reduced fetal gene expression levels in the AKO to below those of littermate WT controls.

## Discussion

We have previously hypothesized that different forms of myocardial stress result in a differential induction of pathways that mediate either a physiologic, reversible, adaptive hypertrophic response (e.g., exercise, pregnancy), or a pathologic, maladaptive hypertrophic response that inevitably leads to decompensatory remodeling and ventricular failure (e.g., volume overload, MI) [2]. We have further postulated that a balance between these molecular regulatory responses within cardiac myocytes determines the fate of the stressed myocardium, and that a therapeutic opportunity therefore exists to affect this balance and shift it toward a physiologic adaptive signaling pattern even under disease conditions.

Previous studies have documented the potential benefit from supraphysiologic alpha1AR gain-of-function during pathologic remodeling after MI and during doxorubicin-induced cardiomyopathy [15, 23]. In the present study, however, we have exploited a unique loss-of-function model to demonstrate the critical physiological role that alpha1AR and downstream MEK-ERK signaling plays in determining the balance between physiologic and pathologic response to stress after MI. In fact, we have furthered the potential therapeutic goal of manipulating this balance via documentation of the role that alpha1-adrenergic signaling, associated in the past with adaptive hypertrophy, likely plays in ameliorating the negative impact of a maladaptive response to the pathologic stresses of the post-MI myocardium. In the absence of MI, the AKO demonstrated normal cardiac function and physiological characteristics. However, there was a significant increase in phospho-p38 (23-fold), phospho-MEK1 (1.4-fold) and MKP1 (1.8-folds). Coupled with a numeric but non-significant increase in echocardiographic measurement of LV mass, these data suggest a possible compensatory increase in pathologic hypertrophy even without MI in the absence of alpha1AR signaling.

ACE inhibition has been well documented to dampen pathologic hypertrophy through its modulation of the rennin-angiotensin system. We therefore treated AKO mice and their littermate WT controls with captopril to further test our hypothesis that the loss of alpha1AR-mediated adaptive hypertrophy led to a corresponding increase in pathologic hypertrophy in response to the various stresses experienced by the post-MI myocardium. Although ACE inhibition did have an expected ameliorative effect on WT hearts, in general the physiologic, anatomic and clinical benefits were substantially increased in the AKO mice, suggesting that pathologic hypertrophy had, in fact, played a more prominent role in their manifestation of post-MI remodeling.

It has been known for some time that specific loss of alpha adrenergic signaling can contribute to human cardiac pathophysiology. The ALHAT and V-HeFT studies, for example, indicated that alpha adrenergic blockade worsens outcomes in the clinical contexts of hypertension and heart failure, respectively [24,25]. Baseline changes in molecular signaling in the AKO mice, even prior to MI, including a substantial increase in the pro-inflammatory and pro-apoptotic regulator phopho-p38 [21,26], might have predisposed the AKO myocardium to stress-induced injury. MI induced both phosphorylation of MEK1 (ERK1/2 activator) but also an increase in the ERK1/2-inactivating phosphatase MKP3; as a result we did not observe an increase in ERK1/2 activation in the post-MI, remote, non-infarcted WT myocardium. ERK1/2 phosphorylation, however, was significantly lower post-MI in AKO than in WT, as was the phospho-ERK1/2:total ERK1/2 ratio.

These changes in MAPK signaling pathways in the AKO myocardium suggest that alpha1AR may contribute to the regulation and amelioration of cardiac responses under stress. In fact, loss of just the specific alpha1A adrenergic subtype significantly worsened ventricular pathologic remodeling via a paradoxical increase in pathologic hypertrophy and subsequent dilatation. Loss of the alpha1A-AR also led to an increase in apoptosis and fibrosis of the uninfarcted remote myocardium, and resulted in substantially higher post-MI mortality. These findings corroborate our hypothesized role of a balance between pro-adaptive and maladaptive signaling in determining the course of post-MI remodeling, and lay a stronger foundation for seeking therapeutic alteration of this balance as a novel approach to preventing heart failure after MI.

## Supporting information

S1 File Western blots. Original blots used in Figs 2 and 4.
(PPT)

S2 File Echocardiography data. Measurements captured from each mouse which were used to create Table 1.
(XLSX)

S3 File Anatomical measurements. Anatomical measurements captured from each mouse which were used to create Table 1.
(XLSX)

## References

[1] Yeh C-C, Malhotra D, Yang Y-L, Xu Y, Fan Y, Li H, et al MEK1-induced physiological hypertrophy inhibits chronic post-myocardial infarction remodeling in mice. J Cell Biochem. 2013;114:47–55. doi: 10.1002/jcb.24299 22821618 10.1002/jcb.24299

[2] Yeh CC, Li H, Malhotra D, Turcato S, Nicholas S, Tu R, et al Distinctive ERK and p38 signaling in remote and infarcted myocardium during post-MI remodeling in the mouse. J Cell Biochem. 2010; 109:1185–1191. doi: 10.1002/jcb.22498 20186881 10.1002/jcb.22498 PMC3397305

[3] Badenhorst D, Veliotes D, Maseko M, Tsotetsi OJ, Brooksbank R, Naidoo A, et al Beta-adrenergic activation initiates chamber dilatation in concentric hypertrophy. Hypertension. 2003; 41:499–504. doi: 10.1161/01.HYP.0000056601.29613.DD 12623950 10.1161/01.HYP.0000056601.29613.DD

[4] O'Connell TD, Swigart PM, Rodrigo MC, Ishizaka S, Joho S, Turnbull L, et al Alpha1-adrenergic receptors prevent a maladaptive cardiac response to pressure overload. J Clin Invest. 2006; 116:1005–1015. doi: 10.1172/JCI22811 16585965 10.1172/JCI22811 PMC1421341

[5] Woo SH, Lee CO. Role of PKC in the effects of alpha1-adrenergic stimulation on Ca2+ transients, contraction and Ca2+ current in guinea-pig ventricular myocytes. Pflugers Arch. 1999; 437:335–344. 9914389 10.1007/s004240050787

[6] Hosoda C, Koshimizu TA, Tanoue A, Nasa Y, Oikawa R, Tomabechi T, et al Two alpha1-adrenergic receptor subtypes regulating the vasopressor response have differential roles in blood pressure regulation. Mol Pharmacol. 2005; 67:912–922. doi: 10.1124/mol.104.007500 15598970 10.1124/mol.104.007500

[7] Hu ZW, Shi XY, Lin RZ, Chen J, Hoffman BB. alpha1-Adrenergic receptor stimulation of mitogenesis in human vascular smooth muscle cells: role of tyrosine protein kinases and calcium in activation of mitogen-activated protein kinase. J Pharmacol Exp Ther. 1999; 290:28–37. 10381756

[8] McCloskey DT, Rokosh DG, O'Connell TD, Keung EC, Simpson PC, Baker AJ. 1-Adrenoceptor Subtypes Mediate Negative Inotropy in Myocardium from alpha 1 A/C-Knockout and Wild Type Mice. J Mol Cell Cardiol. 2002; 34:1007–1017. 12234770 10.1006/jmcc.2002.2049

[9] McCloskey DT, Turnbull L, Swigart P, O'Connell TD, Simpson PC, Baker AJ. Abnormal myocardial contraction in alpha(1A)- and alpha(1B)-adrenoceptor double-knockout mice. J Mol Cell Cardiol. 2003; 35:1207–1216. 14519431 10.1016/s0022-2828(03)00227-x

[10] O'Connell TD, Ishizaka S, Nakamura A, Swigart PM, Rodrigo MC, Simpson GL, et al The alpha(1A/C)- and alpha(1B)-adrenergic receptors are required for physiological cardiac hypertrophy in the double-knockout mouse. J Clin Invest. 2003; 111:1783–1791. doi: 10.1172/JCI16100 12782680 10.1172/JCI16100 PMC156101

[11] Rorabaugh BR, Ross SA, Gaivin RJ, Papay RS, McCune DF, Simpson PC, et al alpha1A- but not alpha1B-adrenergic receptors precondition the ischemic heart by a staurosporine-sensitive, chelerythrine-insensitive mechanism. Cardiovasc Res. 2005; 65:436–445. doi: 10.1016/j.cardiores.2004.10.009 15639483 10.1016/j.cardiores.2004.10.009

[12] Mani K, Ashton AW, Kitsis RN. Taking the BAD out of adrenergic stimulation. J Mol Cell Cardiol. 2002; 34:709–912. 12099710 10.1006/jmcc.2002.2042

[13] Huang Y, Wright CD, Merkwan CL, Baye NL, Liang Q, Simpson PC, et al An alpha1A-adrenergic-extracellular signal-regulated kinase survival signaling pathway in cardiac myocytes. Circulation. 2007; 115:763–772. doi: 10.1161/CIRCULATIONAHA.106.664862 17283256 10.1161/CIRCULATIONAHA.106.664862

[14] Du XJ, Fang L, Gao XM, Kiriazis H, Feng X, Hotchkin E, et al Genetic enhancement of ventricular contractility protects against pressure-overload-induced cardiac dysfunction. J Mol Cell Cardiol. 2004; 37:979–987. doi: 10.1016/j.yjmcc.2004.07.010 15522275 10.1016/j.yjmcc.2004.07.010

[15] Du XJ, Gao XM, Kiriazis H, Moore XL, Ming Z, Su Y, et al Transgenic alpha1A-adrenergic activation limits post-infarct ventricular remodeling and dysfunction and improves survival. Cardiovasc Res. 2006; 71:735–743. doi: 10.1016/j.cardiores.2006.06.015 16859660 10.1016/j.cardiores.2006.06.015

[16] Ren J, Zhang S, Kovacs A, Wang Y, Muslin AJ. Role of p38alpha MAPK in cardiac apoptosis and remodeling after myocardial infarction. J Mol Cell Cardiol. 2005; 38:617–623. doi: 10.1016/j.yjmcc.2005.01.012 15808838 10.1016/j.yjmcc.2005.01.012

[17] Qin F, Liang MC, Liang CS. Progressive left ventricular remodeling, myocyte apoptosis, and protein signaling cascades after myocardial infarction in rabbits. Biochim Biophys Acta. 2005; 1740:499–513. doi: 10.1016/j.bbadis.2004.11.007 15949720 10.1016/j.bbadis.2004.11.007

[18] Huang Y, Wright CD, Kobayashi S, Healy CL, Elgethun M, Cypher A, et al GATA4 is a survival factor in adult cardiac myocytes but is not required for alpha1A-adrenergic receptor survival signaling. Am J Physiol Heart Circ Physiol. 2008; 295:H699–707. doi: 10.1152/ajpheart.01204.2007 18552157 10.1152/ajpheart.01204.2007 PMC2519221

[19] Rokosh DG, Simpson PC. Knockout of the alpha 1A/C-adrenergic receptor subtype:the alpha 1A/C is expressed in resistance arteries and is required to maintain arterial blood pressure. Proc Natl Acad Sci U S A. 2002; 99:9474–9479. doi: 10.1073/pnas.132552699 12093905 10.1073/pnas.132552699 PMC123165

[20] Hosoda C, Hiroyama M, Sanbe A, Birumachi J, Kitamura T, Cotecchia S, et al Blockade of both alpha1A- and alpha1B-adrenergic receptor subtype signaling is required to inhibit neointimal formation in the mouse femoral artery. Am J Physiol Heart Circ Physiol. 2007; 293:H514–519. doi: 10.1152/ajpheart.00626.2006 17384126 10.1152/ajpheart.00626.2006

[21] Ma XL, Kumar S, Gao F, Louden CS, Lopez BL, Christopher TA, et al Inhibition of p38 mitogen-activated protein kinase decreases cardiomyocyte apoptosis and improves cardiac function after myocardial ischemia and reperfusion. Circulation. 1999; 99:1685–1691. 10190877 10.1161/01.cir.99.13.1685

[22] Kajstura J, Cheng W, Reiss K, Clark WA, Sonnenblick EH, Krajewski S, et al Apoptotic and necrotic myocyte cell deaths are independent contributing variables of infarct size in rats. Lab Invest. 1996; 74:86–107. 8569201

[23] Montgomery MD, Chan T, Swigart PM, Myagmar BE, Dash R, Simpson PC. An alpha-1a adrenergic receptor agonist prevents acute doxorubicin cardiomyopathy in male mice. *PLoS One*. 2017;12:e0168409 doi: 10.1371/journal.pone.0168409 28081170 10.1371/journal.pone.0168409 PMC5231318

[24] ALLHAT CRG. Major cardiovascular events in hypertensive patients randomized to doxazosin vs chlorthalidone: The antihypertensive and lipid-lowering treatment to prevent heart attack trial (allhat). [see comments]. *JAMA*. 2000;283:1967–1975. 10789664

[25] Cohn JN. The vasodilator-heart failure trials (v-heft). Mechanistic data from the va cooperative studies. Introduction. *Circulation*. 1993;87:VI1–4. 8500232

[26] Frantz S, Behr T, Hu K, Fraccarollo D, Strotmann J, Goldberg E, et al Role of p38 mitogen-activated protein kinase in cardiac remodelling. Br J Pharmacol. 2007 1;150(2):130–5. doi: 10.1038/sj.bjp.0706963 ; PubMed Central PMCID: PMC2042905. 17179956 10.1038/sj.bjp.0706963 PMC2042905

