# Supplementary figures and images for: Shift toward greater pathologic post-myocardial infarction remodeling with loss of the adaptive hypertrophic signaling of alpha1 adrenergic receptors in mice
Source: PLoS One. 2017 Dec 7;12(12):e0188471. doi: 10.1371/journal.pone.0188471 (PMC5720786; doi:10.1371/journal.pone.0188471)

## Slide 1
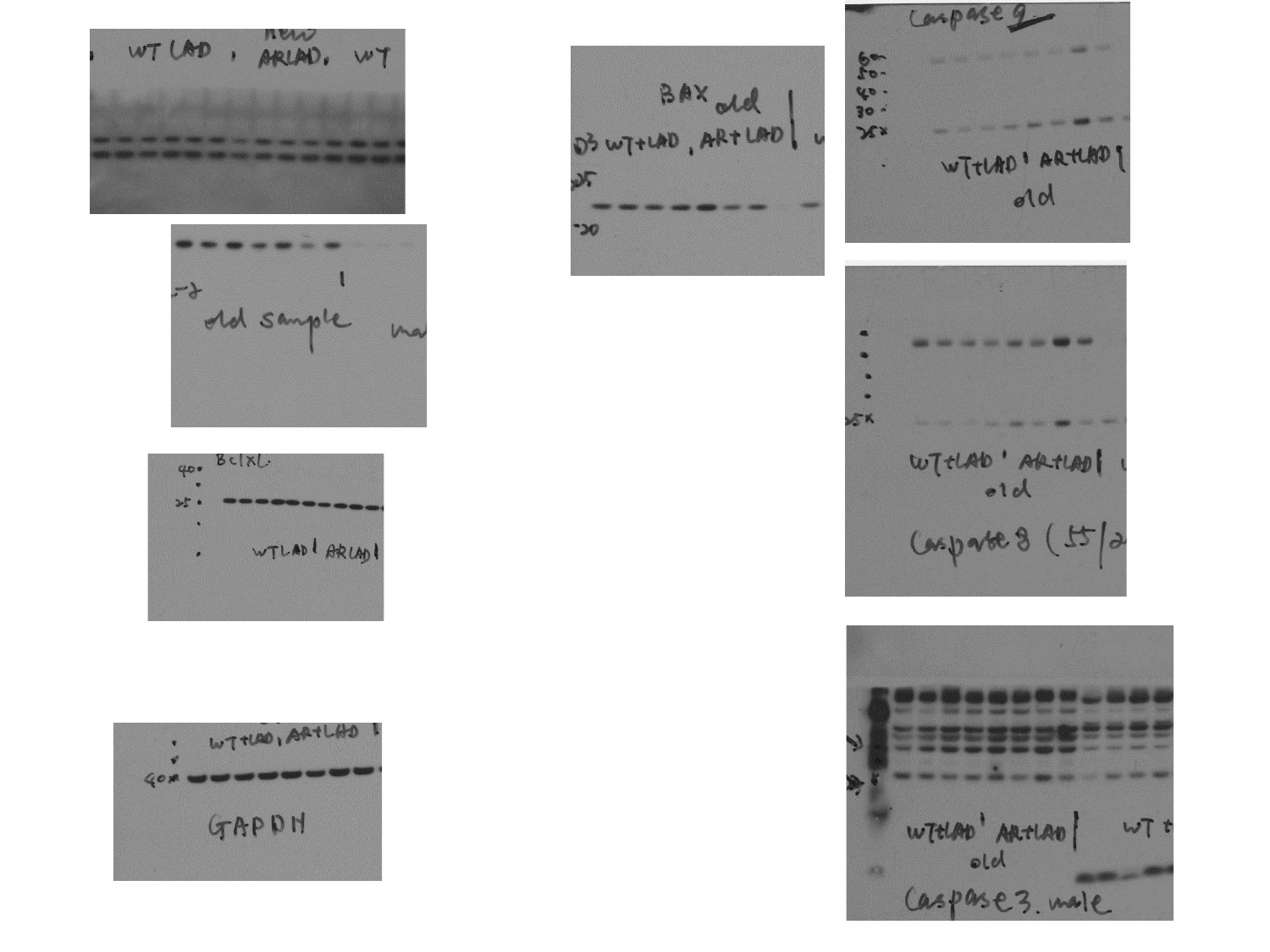

## Slide 2
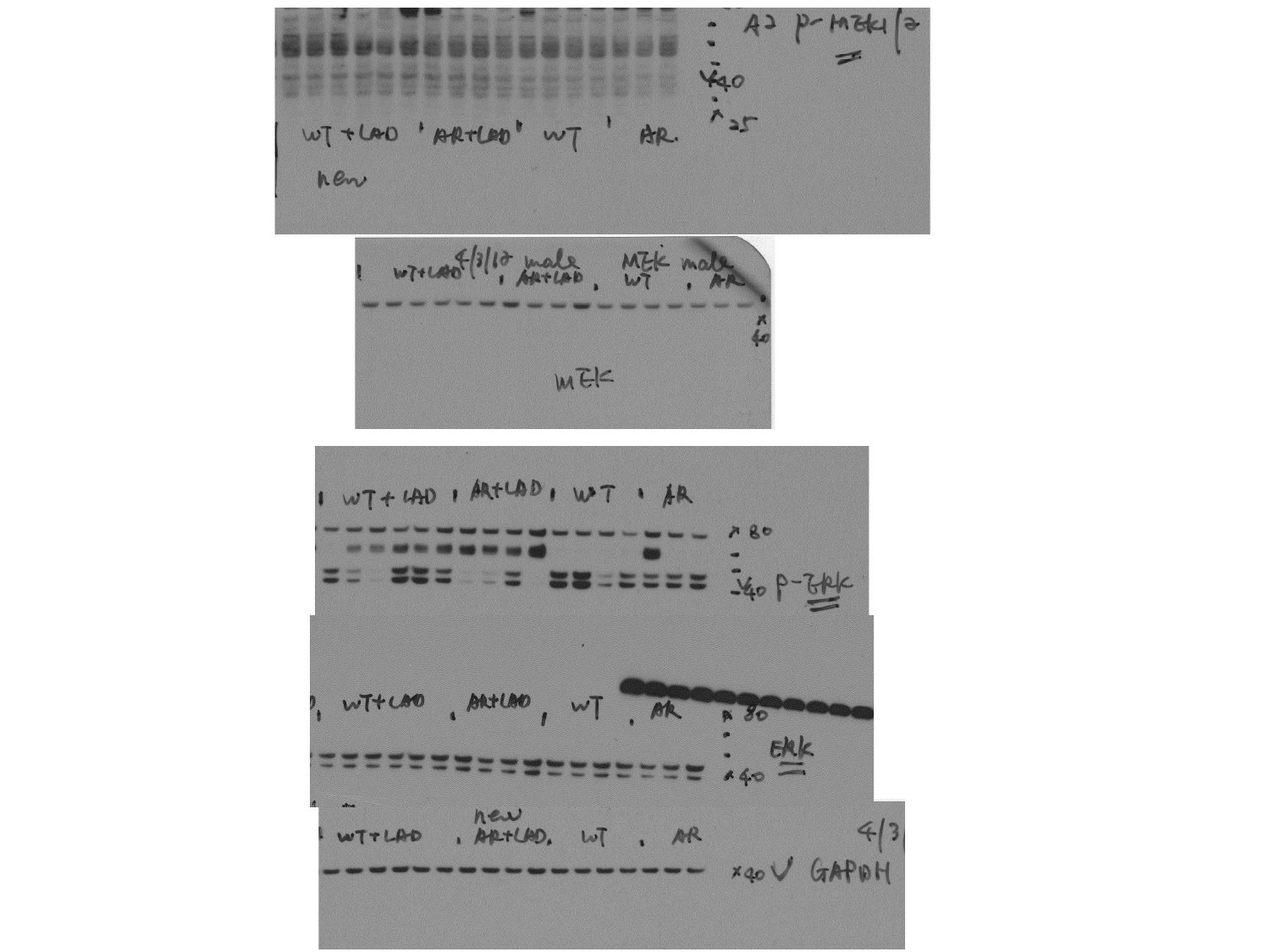

## Slide 3
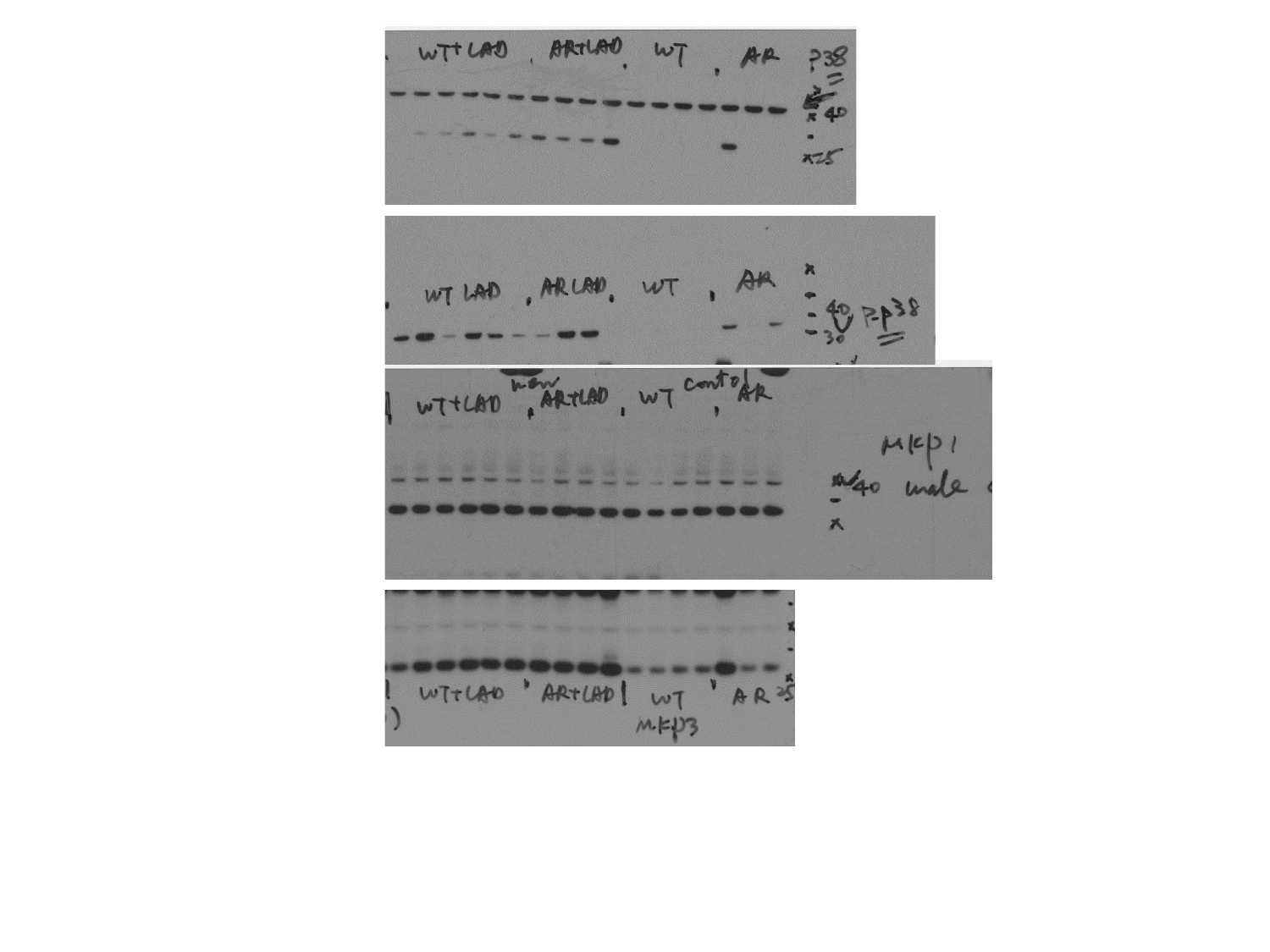

Supplement: S1 File — Original blots used in Figs 2 and 4. (PPT) [file pone.0188471.s001.ppt]
